# Connective Tissue Disorder-Induced Diffuse Alveolar Hemorrhage: A Comprehensive Review with an Emphasis on Airway and Respiratory Management

**DOI:** 10.3390/life15050793

**Published:** 2025-05-15

**Authors:** Mayuri Mudgal, Swetha Balaji, Ajeetha Priya Gajendiran, Ananthraj Subramanya, Shanjai Krishnan Murugan, Venkatesh Gondhi, Aseem Rai Bhatnagar, Kulothungan Gunasekaran

**Affiliations:** 1 Internal Medicine, Camden Clark Medical Center, Parkersburg, WV 26101, USA; 2 Internal Medicine, St. Mary’s General Hospital, New York Medical College and St. Clare’s Health, Passaic, NJ 07055, USA; n.b.swetha@gmail.com; 3 Pulmonary and Critical Care, Onvida Health, Yuma, AZ 85364, USA; ajeetha19@gmail.com (A.P.G.); kgunasekaran@yumaregional.org (K.G.); 4 Internal Medicine, Onvida Health, Yuma, AZ 85364, USA; ananthraj.s91@gmail.com; 5 Department of Medicine, Stanley Medical College, Chennai 600001, TN, India; shanjaikrishnan@gmail.com; 6 Hospital Medicine, Onvida Health, Yuma, AZ 85364, USA; gondhivenkatesh@gmail.com; 7 Department of Radiation Oncology, Henry Ford Health System, Detroit, MI 48202, USA; abhatna1@hfhs.org

**Keywords:** connective tissue disorder, diffuse alveolar hemorrhage, bronchoalveolar lavage, invasive ventilation, airway assessment, corticosteroids, lung ultrasonography, extracorporeal membrane oxygenation

## Abstract

Diffuse alveolar hemorrhage (DAH), a catastrophic complication of connective tissue disorders (CTDs), manifests as rapid-onset hypoxemia, alveolar infiltrates, and progressive bleeding into the airways. While immune-mediated alveolar–endothelial injury primarily drives its pathophysiology, diagnosis is based on bronchoscopy and chest imaging. The clinical urgency lies in securing the compromised airway and stabilizing respiratory failure, a challenge increased by CTD-specific anatomical alterations such as cervical spine instability, cricoarytenoid arthritis, and subglottic stenosis. High-dose corticosteroids and immunosuppression are essential, while severe cases require extracorporeal membrane oxygenation or plasmapheresis. This comprehensive review introduces two novel approaches to address fundamental gaps in the management of CTD-induced DAH: a structured algorithm for a CTD-specific airway risk stratification tool, integrating anatomical screening and the application of lung ultrasounds (LUSs) for post-intubation CTD-induced DAH ventilation management. The need for a multidisciplinary team approach is also discussed. Despite aggressive care, mortality remains high (25–50%), underscoring the necessity for improved early recognition and intervention strategies for these high-risk patients.

## 1. Introduction

Diffuse alveolar hemorrhage (DAH) is a condition characterized by the extravasation of blood into the alveolar spaces of the lungs, potentially leading to respiratory failure [1,2]. DAH is fatal due to the presence of hemoptysis, anemia, diffuse alveolar infiltrates, and rapid patient decompensation. DAH can be attributed to various reasons, including autoimmune illnesses, coagulation abnormalities, medications, inhaled toxins, and transplantation. Immunological disorders constitute 30–40% of all causes of DAH [3]. In patients with autoimmune diseases, primarily connective tissue disorders (CTDs), DAH constitutes around 12% of intensive care unit (ICU) admissions, frequently requiring mechanical ventilation due to significant respiratory impairment. Bronchoscopy with bronchoalveolar lavage (BAL) confirms the diagnosis of diffuse alveolar hemorrhage and excludes infections [2,3,4,5].

This comprehensive review addresses two critical gaps in the management of CTD-induced DAH. First, it proposes a novel airway risk stratification system to identify CTD-induced DAH patients requiring airway intervention—a critical unmet need, as current practice relies on clinical judgment alone. Second, while lung ultrasound (LUS) is well-validated for managing acute respiratory distress syndrome (ARDS), its application to CTD-induced DAH remains unexplored, particularly for guiding ventilation strategies. In this manuscript, we hypothesize a post-intubation LUS-guided positive end-expiratory pressure (PEEP) titration framework adapted from ARDS criteria but modified for CTD-induced DAH’s distinct pathophysiology. Together, these complementary approaches offer a structured pathway for managing this high-mortality complication.

Finally, managing CTD-induced DAH requires an integrated and coordinated multidisciplinary team strategy to address the challenges of life-threatening respiratory failure and underlying immune dysregulation.

## 2. Pathophysiology

**Figure 1:**
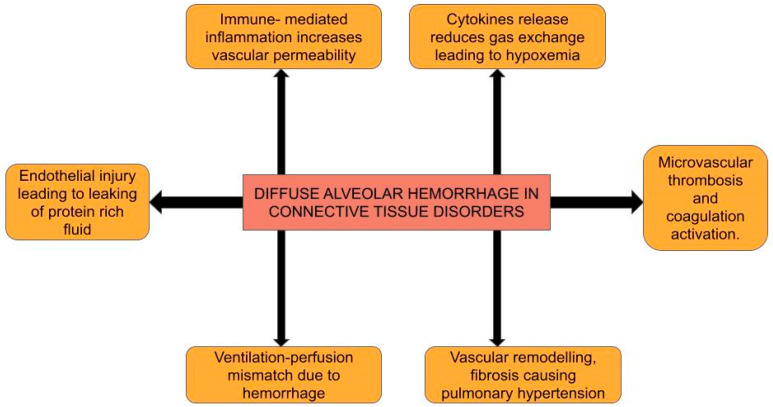
Illustration of the varied pathophysiological mechanisms of CTD-induced DAH.

Various pathophysiological mechanisms contribute to the widespread alveolar hemorrhage associated with CTDs. These mechanisms are outlined below (Figure 1).

### 2.1. Immune Complex-Mediated Endothelial Injury

The main cause of CTD-induced DAH is immune-mediated inflammation. Systemic lupus erythematosus (SLE) and anti-neutrophil cytoplasmic antibody (ANCA)-associated vasculitis often begin with immune complexes on pulmonary capillaries. These complexes activate the classical complement system, producing C3a and C5a inflammatory mediators that signal neutrophil chemotaxis and activation. Activated neutrophils release ROS and proteolytic enzymes, including elastase and myeloperoxidase, causing capillaritis and alveolar damage [1,2].

### 2.2. Cytokine Release, Signal Transduction, and Endothelial Barrier Disruption

Lung architecture and function depend on the alveolar–capillary barrier. Cytokines, such as IL-1β, IL-6, and TNF-α, enhance the inflammatory cascade by altering endothelial tight junction expression and permeability [3,4,5]. Red blood cells, inflammatory cells, and protein-rich fluid pass through the alveolar interstices due to enhanced permeability. In addition, Zhuang et al. have noted that in pristane-treated lupus mouse models, activation of the MEK1/2–ERK1/2 signaling pathway occurs, which leads to endothelial cell damage and thrombosis [6]. Hemostasis impairment from this signaling cascade causes microvascular bleeding into the alveoli [6]. This leads to the impairment of gas exchange, causing hypoxemia [7,8,9,10,11].

### 2.3. Macrophage Activation and Polarization

In murine DAH models, bone marrow-derived monocytes/macrophages in the lungs are critical for alveolar hemorrhage, and their enhanced pro-inflammatory M1 polarization is the key immunological mechanism [12,13,14]. The activation of M1 macrophages via NF-κB and MAPK pathways leads to the release of pro-inflammatory cytokines, including TNFα, IL-1β, IL-6, IL-12, IL-15, and IL-23, which generate cytotoxic reactive oxygen and nitrogen species (ROS and RNS, respectively). ROS and RNS directly damage the alveolar–capillary membrane, causing acute lung injury [15].

### 2.4. Chronic Inflammation, Vascular Remodeling, and Fibrosis

CTDs can cause chronic inflammation and vascular remodeling. Human and animal studies show that endothelial dysfunction causes fibrosis, vascular thickening, and pulmonary hypertension [8,9,11]. If untreated, this can lead to persistent respiratory failure. Collagen deposition and intimal hyperplasia have been observed in pulmonary vasculopathy associated with lupus and scleroderma [8,9,11].

## 3. Clinical Presentation

CTD-induced DAH is an emergent condition that requires urgent intervention. The amount of bleeding in the alveoli, the type of connective tissue illness, and the involvement of other systems influence its clinical presentation.

### 3.1. Classic Clinical Triad

1. Hemoptysis occurs in 60–70% of cases; however, it may be absent in up to one-third of patients, particularly in those with severe anemia or non-massive hemorrhage [2]. Hemoptysis must be differentiated from hematemesis or pseudo-hemoptysis (denoting alveolar flooding that resembles blood, as noted in Serratia marcescens pneumonia, in which the reddish hue of the organism creates an impression of alveolar bleeding [16]).
2. Anemia, either normochromic or due to iron deficiency, is characterized by a rapid decline in hemoglobin levels. It is frequently disproportionate to external blood loss [17].
3. Diffuse pulmonary infiltrates are characterized by the presence of abnormal substances within the lung interstitium, often indicative of underlying pathological processes. Bilateral ground-glass opacities or consolidations observed in chest imaging indicate alveolar filling [2,17,18].

Importantly, the presentation of DAH in CTDs is often complicated by systemic manifestations of the underlying autoimmune disease, which may inform clinical suspicion and diagnosis.

### 3.2. Respiratory Symptoms

1. Dyspnea: This is the most prevalent symptom, progressing quickly to respiratory failure [17].
2. Cough: Typically non-productive or blood-tinged; however, significant hemoptysis can occur [18].
3. Hypoxia: Severe cases result in acute respiratory distress syndrome (ARDS), necessitating high-flow nasal oxygen (HFNC) or mechanical ventilation [2,17,18].

### 3.3. Systemic Characteristics of CTDs Associated with DAH

Systemic autoimmune involvement can provide important diagnostic clues for DAH. The features of systemic and pulmonary involvement in CTD-induced DAH are summarized in Supplementary Table S1.

1. Systemic Lupus Erythematosus (SLE)
  - DAH develops in up to 11% of patients with SLE [16].
  - Lupus nephritis, fever, arthritis, and anti-dsDNA antibody positivity have all been linked [18,19].
2. Granulomatosis with Polyangiitis (GPA; originally Wegener’s)
  - Common cause of DAH, presenting with recurrent sinusitis, epistaxis, nasal crusting, and kidney involvement [20,21].
3. Microscopic Polyangiitis (MPA)
  - Pulmonary–renal disease is characterized by ANCA-positive results, along with rapidly progressing glomerulonephritis and alveolar bleeding [22,23].
4. Rheumatoid Arthritis (RA)
  - RA can cause severe DAH, especially in patients with secondary vasculitis or on immunosuppressive therapy [24,25].
5. Anti-phospholipid Syndrome (APS)
  - Anti-phospholipid syndrome (APS) is characterized by frequent thrombosis, livedo reticularis, and pregnancy loss [26,27].
  - APS-related DAH is frequently associated with catastrophic APS and microvascular thrombosis [26,27].
6. Scleroderma (Systemic Sclerosis)
  - While interstitial lung disease is more common, DAH can arise in the presence of renal crisis or pulmonary hypertension [28].
7. Polymyositis and Dermatomyositis (PM-DM)
  - Pulmonary involvement precedes the muscular manifestations of PM-DM by many years or occurs simultaneously [29].
8. Mixed connective tissue disorder
  - Approximately 73% of patients have pulmonary involvement, with few developing DAH [30].

## 4. Radiographic and Diagnostic Features of DAH in Connective Tissue Disorders

CTD-induced DAH diagnosis integrates comprehensive clinical evaluations, serologic testing for CTDs, and bronchoscopic and radiographic findings.

### 4.1. Chest X-Ray (CXR)

**Figure 2:**
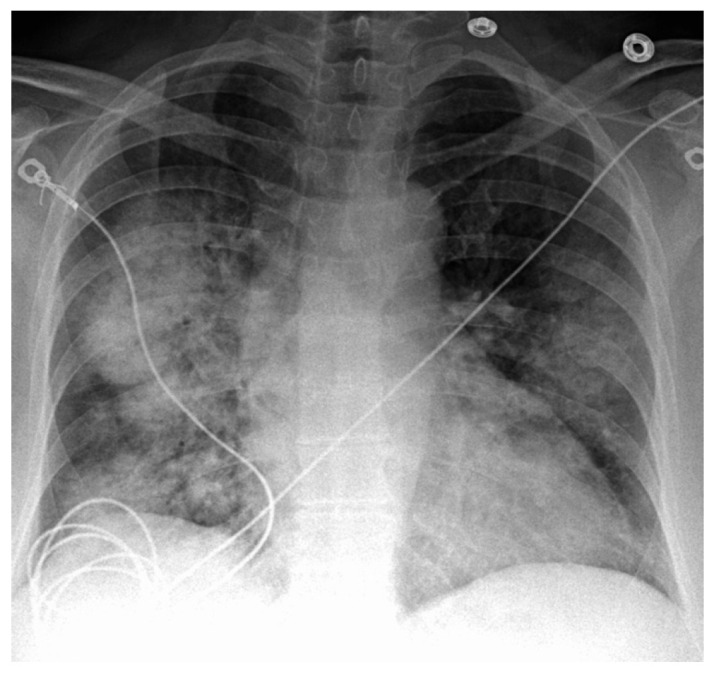
Chest X-ray showing asymmetric, diffuse, bilateral heterogenous airspace disease and consolidations in a patient with SLE, consistent with DAH. (This work has been reproduced from Jamsheer et al., 2024, in accordance with the terms of the Creative Commons Attribution License [32]).

This displays diffuse bilateral airspace opacities, typically in a central or perihilar distribution (Figure 2). Since these findings are quite non-specific, they may resemble pulmonary edema, pneumonia, or acute respiratory distress syndrome (ARDS) [16,31,32,33]. Furthermore, a CXR may appear normal in early or mild instances [34].

### 4.2. High-Resolution CT (HRCT) of the Chest

**Figure 3:**
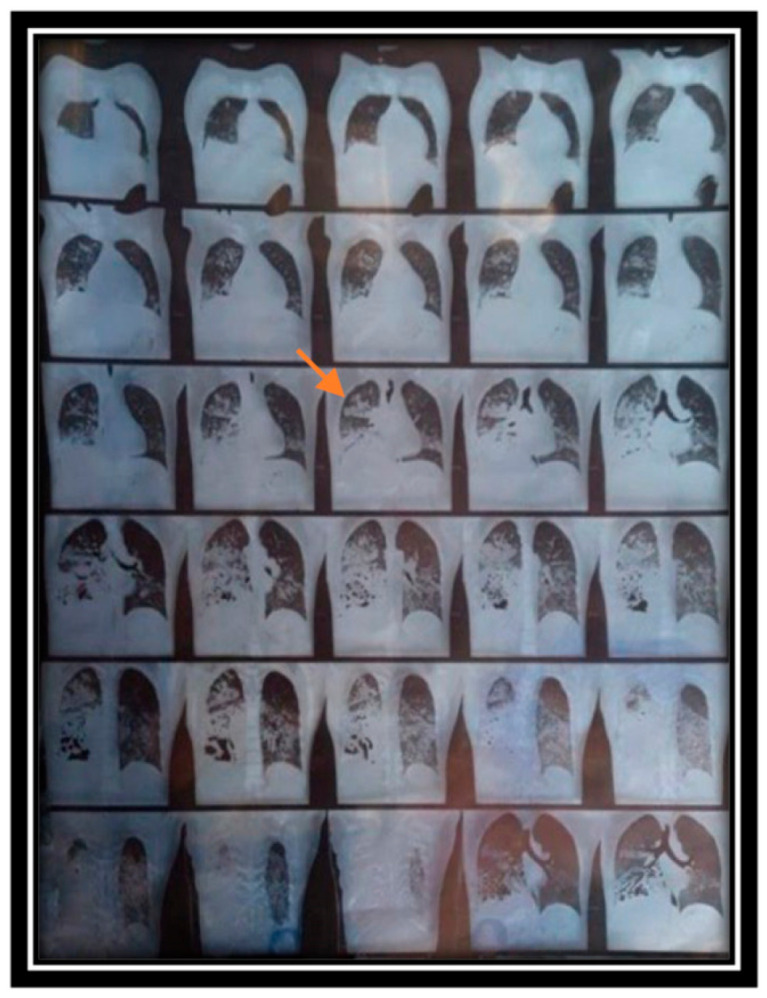
Chest CT of a patient with diffuse alveolar hemorrhage due to granulomatosis with polyangiitis, depicting worsening bilateral ground-glass opacities with areas of consolidation indicated by the orange arrow. (This work has been reproduced from Clinical Case Reports, 2024, under the terms of the Creative Commons Attribution License [35]).

An HRCT reveals ground-glass opacities and alveolar consolidations, predominantly located in dependent lung areas due to fluid buildup(Figure 3) [35]. Interstitial thickening may occur in underlying connective tissue disease-associated interstitial lung disease (ILD) [36].

Since there are no notable septal thickening or pleural effusions, it can aid in distinguishing it from pulmonary edema. Serial imaging can also demonstrate the swift resolution of opacities alongside the clearance of bleeding [2,36].

### 4.3. Bronchoalveolar Lavage (BAL)

Bronchoscopy is crucial for confirming diffuse alveolar hemorrhage (DAH) and excluding viral, cardiogenic, or neoplastic etiologies. Progressive hemorrhagic return (increasing blood content in successive lavage aliquots) strongly indicates DAH [37].

**Figure 4:**
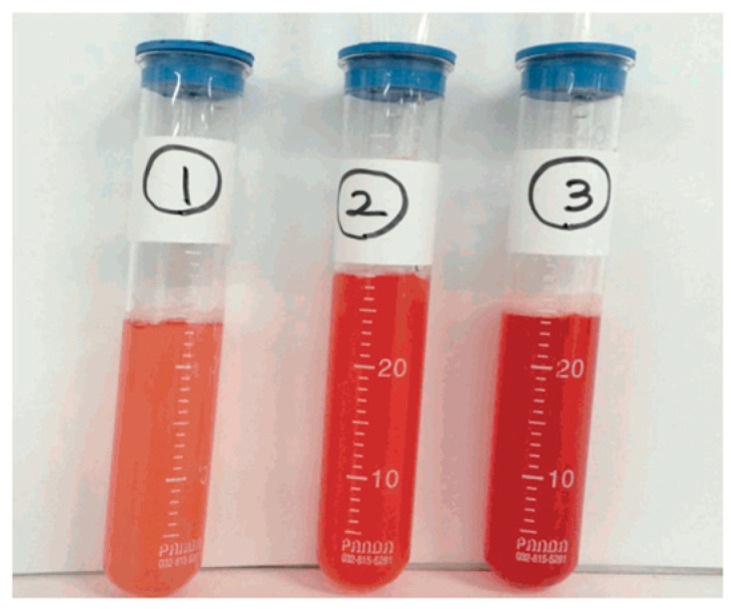
BAL showing serial aliquots of increasing amounts of red blood cells, confirming the presence of DAH. (This work has been reproduced from Moon KM et al. under the terms of the Creative Commons Attribution License [40]).

Bronchoalveolar lavage cytology may show macrophages filled with hemosiderin, which appear 24–48 h after bleeding starts and can last for several weeks. Therefore, it has a higher yield if performed in the first 48 h. Usually, the presence of ≥20% hemosiderin-laden macrophages in the BAL fluid is considered diagnostic of DAH and is closely associated with severity (Figure 4) [37,38,39]. Sending BAL specimens for routine bacterial, mycobacterial, fungal, and viral cultures, as well as Pneumocystis stains, assists in excluding infections [2].

### 4.4. Pulmonary Function Tests (PFTs)

In less acute settings and when the patient is stable, pulmonary function tests could also help in the early identification of DAH. An increase in the diffusing capacity of the lung for carbon monoxide (DLCO) is the characteristic finding, which is secondary to the increased presence of hemoglobin in the airspaces, causing the removal of carbon monoxide from exhaled air [41].

### 4.5. Autoimmune Serological Analysis

Routine lab work, including a complete blood count with differentials, a chemistry panel, liver function tests, blood urea nitrogen and creatinine levels, a coagulation panel (PT, aPTT, INR, and D-dimer), and urine analysis with microscopic examination for proteinuria and microscopic hematuria, provides useful initial insights. Table 1 highlights the specific and non-specific labs that assist with diagnosing some CTDs leading to DAH.

CTDs are associated with elevated erythrocyte sedimentation rate (ESR) and C-reactive protein (CRP) levels [42]. Serum ANCA, ANA, rheumatoid factor (RF), and anti-phospholipid antibodies should also be obtained along with dsDNA, anti-ribonucleoprotein, and anti-JO-1 [2]. The p-ANCA pattern is observed in antibodies directed against a variety of intracellular antigens, most commonly with myeloperoxidase (MPO). C-ANCA is highly sensitive (90–95%) in active, systemic granulomatosis with polyangiitis, with a specificity of approximately 90% [2]. The anti-cardiolipin antibody would aid in identifying DAH due to anti-phospholipid antibody syndrome [43].

### 4.6. Histopathological Examination

The role of a transbronchial lung biopsy in the diagnosis of DAH is not fully established, primarily because the areas of involvement in DAH are often patchy and may not be reliably sampled during the procedure [16]. However, in patients presenting with DAH of unclear etiology, a surgical lung biopsy is strongly recommended to help identify the underlying cause and guide appropriate management [44,45,46]. Histopathological examination notes pulmonary capillaritis in ANCA-associated vasculitis, lupus, and anti-phospholipid syndrome (APS), bland hemorrhage in SLE-absent active vasculitis, and interstitial fibrosis in people with underlying interstitial lung disease (ILD) [2]. A skin biopsy in the presence of rashes or a renal biopsy in the setting of glomerulonephritis may also be beneficial in aiding diagnosis [2].

## 5. Management

Management involves supportive, therapeutic, and respiratory support, as discussed below.

### 5.1. Supportive Care

**Figure 5:**
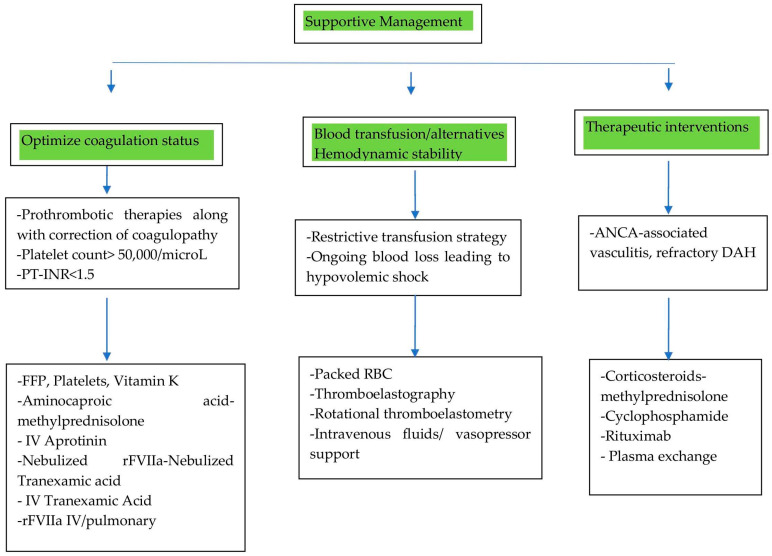
Supportive management for CTD-induced DAH. Abbreviations: RBC: red blood cell; FFP: fresh-frozen plasma; IV: intravenous; rFVIIa: activated recombinant factor VII; PT/INR: prothrombin time/international normalized ratio.

Supportive care in CTD-induced DAH focuses on optimizing coagulation status, managing blood transfusions, and ensuring hemodynamic stability. This scenario is illustrated in Figure 5.

#### 5.1.1. Optimizing Coagulation Status

Generally, in drug-induced diffuse alveolar hemorrhage (DAH), the primary intervention is to discontinue the offending drug, such as anticoagulants, thrombolytic agents, or antiplatelet agents [47]. However, in DAH caused by CTDs, management focuses on standard therapies aimed at controlling bleeding and addressing the underlying autoimmune process. These therapies include fresh-frozen plasma (FFP) and packed red cell (pRBC) transfusions to correct coagulopathy and anemia, respectively, as well as intravenous infusions of aprotinin and tranexamic acid (administered intravenously or via the endotracheal route) to promote hemostasis.

A study by Lars et al. demonstrated that standard therapies often have limited or insufficient hemostatic effects compared to activated recombinant factor VII (rFVIIa) [48,49]. Local intrapulmonary administration of rFVIIa (at a dose of 50 μg/kg via bronchoalveolar lavage) showed superior hemostatic efficacy compared to the intravenous route. This approach reduced the risk of systemic complications in both children and adults [50]. However, it is important to note that local rFVIIa treatment carries a potential risk of inducing ARDS [48].

Combination therapies have also shown efficacy. Aminocaproic acid and methylprednisolone have shown significant reductions in DAH-associated mortality compared to methylprednisolone alone [51]. Nebulized tranexamic acid alone can lead to the cessation of bleeding in many DAH patients. When combined with nebulized rFVIIa, it results in complete hemostasis in most cases [51].

Alongside the use of prothrombotic therapies as mentioned above, it is essential to closely monitor and correct coagulopathy. The following targets are commonly recommended: platelet counts > 50,000/μL and a prothrombin time–international normalized ratio (PT-INR) < 1.5, which can be achieved through platelet and FFP transfusions, as well as vitamin K administration. Maintaining these parameters can minimize the risk of ongoing hemorrhage, support the effectiveness of prothrombotic therapies, and improve overall outcomes in DAH patients [50].

#### 5.1.2. Managing Blood Transfusions: Strategies and Alternatives

While packed red blood cell (pRBC) transfusions can correct anemia in patients with DAH, they do not significantly improve tissue oxygen delivery or oxygen consumption [51]. In hemodynamically stable pediatric DAH patients, a restrictive transfusion strategy—using Lacroix’s threshold of 7 g/dL—is recommended over a liberal approach to minimize risks while effectively managing anemia [51]. However, transfusions are not without significant risks. Both platelet and red blood cell transfusions are independently associated with increased risks of venous and arterial thromboembolism (VTE), as well as in-hospital mortality, particularly in hospitalized oncology patients [52]. Additionally, platelet transfusions carry specific risks, including allergic reactions, transfusion-associated sepsis, and transfusion-related acute lung injury (TRALI) [53].

Given the limited efficacy of traditional transfusions in managing DAH and their associated risks, alternative strategies such as thromboelastography (TEG) and rotational thromboelastometry (ROTEM) are emerging as promising tools. Studies have shown that TEG/ROTEM-guided transfusion in cardiac surgery patients reduces mortality and the requirement for platelet, RBC, and FFP transfusions, thereby suggesting that TEG/ROTEM-guided transfusion could be a valuable approach for managing DAH in the future, offering a more targeted and effective strategy while minimizing risks [54].

#### 5.1.3. Hemodynamic Stability

In the early stages of DAH, patients may remain hemodynamically stable due to compensatory mechanisms such as increased cardiac output and the redistribution of blood volume. However, in cases of severe or persistent DAH, particularly in conditions such as CTDs, ongoing blood loss can lead to hypovolemia and shock. Frequent hemodynamic monitoring is therefore essential. If hypotension occurs, intravenous (IV) fluids should be administered cautiously to avoid the risk of pulmonary edema. In cases of refractory shock, the use of vasopressors may be necessary to maintain adequate perfusion. Importantly, since shock is the most important cause for in-hospital mortality among DAH patients, maintaining hemodynamic stability is critical for improving prognosis and overall outcomes [55].

### 5.2. Therapeutic Interventions

The primary intervention involves corticosteroids, which are recognized as a first-line therapy, particularly in ANCA-associated vasculitis and SLE-related DAH, in conjunction with the aforementioned supportive measures. The regimen includes pulse therapy with methylprednisolone, administered through the IV route at a dose of 500–1000 mg daily for 3–5 days, followed by tapering doses of prednisone. Later maintenance is achieved with a transition to oral prednisone (1 mg/kg/day) with gradual tapering [50].

The steroid-sparing approach, incorporating immunosuppressive therapy with cyclophosphamide (CYC), is used for severe, refractory DAH or vasculitis-related DAH. CYC is given as IV pulses (e.g., 500–1000 mg/m^2^ every 2–4 weeks) or orally (1–2 mg/kg/day). The other option is rituximab (RTX), an alternative to cyclophosphamide in ANCA-associated vasculitis. Rituximab is given as IV infusions (375 mg/m^2^ weekly × 4 or 1000 mg IV on days 0 and 14) [50].

Another treatment option is plasma exchange (PLEX), which is particularly beneficial in severe ANCA-associated vasculitis with rapidly progressive glomerulonephritis. However, recent studies, such as the PEXIVAS trial, have questioned its efficacy in improving outcomes for ANCA-associated vasculitis. PLEX is usually administered in 5–7 sessions over 10–14 days [56].

It is therefore crucial to identify and address the underlying cause of DAH to tailor treatments effectively.

### 5.3. Airway Assessment

A comprehensive pre-intubation evaluation is crucial to identify potential challenges in airway management. A detailed clinical history is a cornerstone of airway assessment in DAH. Patients with previous intubation difficulties, hoarseness, or stridor are at higher risk for airway obstruction. Symptoms such as dysphagia and vocal changes may indicate upper airway involvement, which complicates management. Additionally, comorbidities such as connective tissue disorders frequently result in anatomical alterations, including cervical spine instability, cricoarytenoid arthritis, or subglottic stenosis, all of which may increase the difficulty of intubation. The physical examination should focus on identifying predictors of a difficult airway. Limited mouth opening, a small mandible, and restricted neck mobility are critical findings that signal potential challenges during intubation. Observations of respiratory distress, such as accessory muscle use or paradoxical breathing, highlight the urgency of intervention. Simultaneously, the presence of active hemoptysis or significant secretions can obstruct visualization during airway instrumentation and elevate the risk of aspiration [57].

Functional airway assessment tools, such as the modified Mallampati score, thyromental distance, and upper lip bite test, offer valuable insight into the complexity of intubation [58]. In patients with connective tissue disorders, assessment of the cervical spine is particularly important due to its frequent involvement. Imaging studies further augment airway evaluation, with neck CTs or MRIs providing detailed visualizations of structural abnormalities, such as subglottic stenosis or cervical spine instability. Chest imaging, including X-rays and CT scans, helps assess the extent of alveolar involvement and guides decisions on ventilation strategies [58].

**Figure 6:**
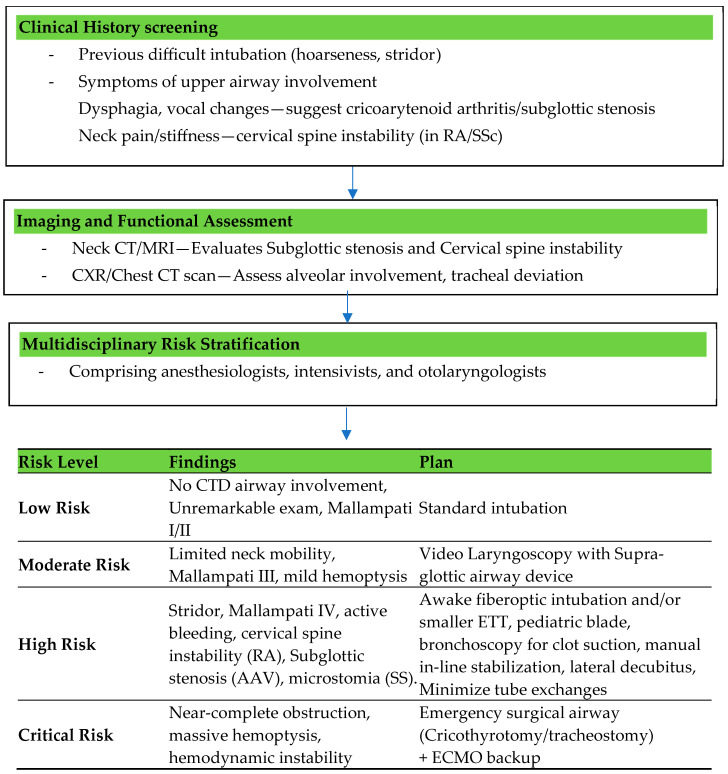
Structured algorithm for airway risk stratification in CTD-induced DAH. Abbreviations: CXR: chest X-ray; ETT: endotracheal tube; ECMO: extracorporeal membrane oxygenation; RA: rheumatoid arthritis; AAV: anti-neutrophil cytoplasmic antibody-associated vasculitis; SS: systemic sclerosis.

Risk stratification is therefore vital for effective planning and should include a multidisciplinary team comprising anesthesiologists, intensivists, and otolaryngologists when necessary. Figure 6 demonstrates the structured algorithm for airway risk stratification in CTD-induced DAH.

#### Challenges in Airway Management Specific to Connective Tissue Disorders

Airway management in patients with connective tissue disorders (CTDs) presents unique challenges due to anatomical and physiological complexities arising from the underlying disease and the acute effects of DAH. CTDs such as systemic lupus erythematosus, scleroderma, and rheumatoid arthritis frequently affect both the upper and lower airways, complicating the management process. Structural abnormalities such as limited temporomandibular joint mobility, cervical spine instability, and cricoarytenoid arthritis significantly increase the likelihood of a difficult airway. Additionally, inflammatory changes and vascular fragility inherent to CTDs increase the risk of mucosal injury during airway manipulation. Pulmonary involvement, such as fibrosis, pleural effusion, pulmonary hypertension, and DAH, necessitates meticulous planning for oxygenation and ventilation [29].

DAH complicates airway assessment due to the critical respiratory compromise induced by alveolar inundation with blood. Airway management in CTD-induced DAH therefore needs careful assessment of the patient’s history, physical examination findings, and advanced imaging to ensure adequate oxygenation and ventilation while avoiding complications [59,60].

Preparation of equipment, including a difficult airway cart with video laryngoscopes, fiberoptic bronchoscopes, and supraglottic airway devices, is imperative to ensure readiness for challenging scenarios. Fiberoptic intubation is often considered the safest approach for patients with an anticipated difficult airway; however, this technique may not be suitable in emergency conditions or when excessive blood or secretions are present in the airway. For tracheal intubation, minimizing neck manipulation with manual in-line stabilization is crucial, even in the absence of overt cervical spine injury. In patients presenting with symptoms of upper airway obstruction or in emergent scenarios where conventional intubation is not feasible, surgical tracheostomy may be indicated [57,58].

Furthermore, the fragility of the airway mucosa in DAH necessitates gentle handling. Pre-oxygenation is critical, and advanced techniques such as high-flow nasal oxygen (HFNO) can be employed to optimize oxygen reserves. Ultimately, the integration of thorough pre-assessment, risk stratification, and advanced planning significantly improves the management of challenging airways in CTD-induced DAH [61].

### 5.4. Oxygenation and Ventilation Strategies

CTD-induced DAH presents as bleeding into the alveolar space, thereby impairing gas exchange and leading to severe hypoxemia and ventilation difficulties [62,63]. The management of oxygenation in CTD-induced DAH requires a carefully tailored approach, balancing the benefits of non-invasive strategies against the risks of delayed intubation and worsening respiratory distress. In patients with mild hypoxemia, conventional oxygen therapy via a nasal cannula or face mask can be sufficient, although close monitoring is required to prevent progression to respiratory failure [17,64].

#### 5.4.1. Indications for Non-Invasive Ventilation

Non-invasive ventilation (NIV) has been explored as a method to improve oxygenation while avoiding the complications of invasive mechanical ventilation in patients with mild to moderate respiratory failure [65]. However, its role in CTD-induced DAH remains controversial due to the complexities of pulmonary involvement, impaired gas exchange, and the risk of worsening alveolar bleeding. NIV (continuous positive airway pressure, CPAP, and bilevel positive airway pressure, BiPAP) can provide temporary respiratory support by reducing the effort of breathing and improving oxygenation [65,66]. It is recommended to use a low to moderate inspiratory positive airway pressure (IPAP) and expiratory positive airway pressure (EPAP) to avoid overdistension while maintaining adequate oxygenation. The fraction of inspired oxygen (FiO_2_) is adjusted to maintain oxygen saturation (SpO_2_) ≥ 92% but avoid excessive oxygenation to prevent hyperoxia-related injury [65,66].

Contraindications for Non-Invasive Ventilation

Absolute contraindications to NIV use include severe facial deformities preventing a proper mask fit, hemodynamic instability, impaired consciousness (making it difficult to protect the airway and increasing the risk of aspiration), and copious secretions [67]. Since DAH can lead to excessive airway secretions and significant hemoptysis, the risk of aspiration and airway obstruction increases, further complicating respiratory support and contraindicating NIV use in patients unable to clear their secretions and maintain airway patency.

Limitations to NIV

In CTD-induced DAH, the presence of ongoing alveolar hemorrhage poses a significant challenge to the use of NIV. The application of positive airway pressure can exacerbate alveolar bleeding by increasing pulmonary capillary pressure, worsening hypoxemia, and potentially leading to ventilator-induced lung injury (VILI). Even in mild-moderate respiratory failure cases, close monitoring is essential, as patients with worsening gas exchange, persistent respiratory distress, or hemodynamic instability may require prompt escalation to invasive mechanical ventilation [68].

#### 5.4.2. Use of High-Flow Nasal Oxygen (HFNO)

Given these challenges, HFNO has emerged as a potential alternative to NIV in CTD-induced DAH. HFNO delivers heated, humidified oxygen at high flow rates, improving oxygenation while reducing respiratory effort without the risks associated with positive-pressure ventilation. It also provides better secretion clearance and enhanced comfort compared to NIV, making it a more suitable option for some patients [61,69]. However, in cases of significant alveolar hemorrhage, progressive respiratory failure, or airway compromise, early invasive mechanical ventilation remains the preferred approach to secure the airway and facilitate lung-protective ventilation strategies.

#### 5.4.3. Indications for Intubation

Timely intubation is critical in patients with CTD-induced DAH to prevent further deterioration [70,71]. Indications include the following:

- Severe hypoxemia, defined as a PaO_2_/FiO_2_ (partial pressure of oxygen in arterial blood by the fraction of inspired oxygen) below 150 despite HFNO or NIV;
- Respiratory failure, characterized by tachypnea greater than 35 breaths per minute, accessory muscle use, paradoxical breathing, or altered mental status due to hypoxia or hypercapnia;
- Hemodynamic instability, including hypotension requiring vasopressor support or worsening shock states secondary to respiratory distress;
- Inability to protect the airway due to altered consciousness, excessive airway bleeding, or progressive upper airway involvement from CTD-associated cricoarytenoid arthritis or subglottic stenosis;
- Failure of non-invasive oxygenation strategies, including NIV intolerance or worsening respiratory parameters despite optimization, prompting consideration for early invasive airway management to prevent further decompensation.

**Invasive Ventilation**

Mechanical ventilatory strategies in CTD-induced DAH should be guided by principles established for ARDS, given the significant overlap in pathophysiology, including alveolar flooding, loss of aerated lung units, and impaired gas exchange [72]. Lung-protective ventilation remains the cornerstone of management, aiming to minimize VILI while ensuring adequate oxygenation and ventilation. Low tidal volume ventilation (4–6 mL/kg of ideal body weight) is recommended to reduce overdistension-related barotrauma and volutrauma, which can further exacerbate alveolar injury [73,74]. Maintaining plateau pressures below 30 cm H_2_O is essential to prevent excessive transpulmonary pressure gradients that could worsen pulmonary hemorrhage. Additionally, the driving pressure (ΔP), which is the difference between the plateau pressure and PEEP, should be kept below 15 cmH2O to optimize lung mechanics and reduce mechanical stress [73,74].

Positive end-expiratory pressure (PEEP) plays a crucial role in alveolar recruitment and oxygenation. However, in CTD-induced DAH, where alveoli are filled with blood and inflammatory exudates, PEEP must be titrated cautiously to balance the benefits of lung recruitment against the risks of hemodynamic compromise and impaired venous return. A high PEEP strategy, often used in ARDS, may not be well-tolerated in this population due to underlying pulmonary hypertension, right ventricular dysfunction, and vascular fragility. Individualized PEEP titration, guided by oxygenation response and hemodynamic stability, is essential to prevent the exacerbation of hemorrhagic lung injury. Permissive hypercapnia is often employed in conjunction with lung-protective ventilation to prevent aggressive ventilatory settings that could exacerbate alveolar injury. However, in patients with comorbidities such as pulmonary hypertension, hypercapnia must be cautiously managed to avoid hemodynamic instability [72]. The subsequent section explores the emerging modality of transthoracic ultrasounds, which may be a valuable tool for addressing concerns regarding optimal PEEP titration in the setting of CTD-induced DAH.

In recent studies, airway pressure release ventilation (APRV) offers benefits by using prolonged high-pressure phases with brief release periods. These short releases help prevent alveolar collapse and VILI by promoting alveolar expansion and improved oxygenation. The maintenance of positive pressure using APRV contributes to the prevention of intra-alveolar bleeding and alleviation of respiratory distress, thereby making it a valuable method for managing respiratory failure in CTD-related DAH [75,76].

Prone positioning has been widely recognized as an effective adjunctive strategy in moderate to severe ARDS, improving ventilation–perfusion matching and reducing shunt fraction by redistributing lung perfusion. In CTD-induced DAH, prone positioning may be beneficial in refractory hypoxemia, but its application must be cautiously considered, given the potential for exacerbating airway bleeding, difficulties in secretion clearance, and challenges in patient positioning due to the musculoskeletal involvement commonly seen in CTDs [77].

**Role of lung ultrasonography in diagnosing ARDS**

**Figure 7:**
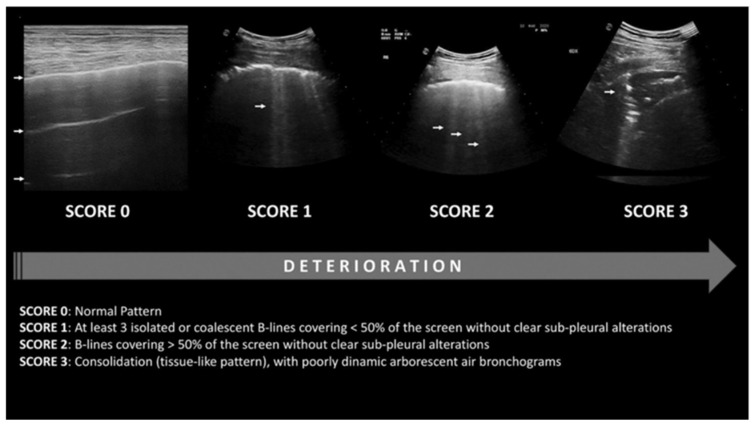
Lung finding for each lung ultrasound score with 12 lung fields. Lung regions exhibiting normal aeration (N) are assigned a score of 0 (white arrows with score 0 denoting A lines); moderate aeration loss (B1) receives a score of 1 (white arrows with score 1 denoting B lines); severe aeration loss (B2) is given a score of 2 (white arrows with score 2 denoting B lines covering > 50% of the screen); and full aeration loss (consolidation; C) is allocated a score of 3 (white arrows denoting consolidation). The cumulative readings for all 12 regions yield a LUS score ranging from 0 to 36. The figure is reproduced from Dell’Aquila et al. 2022 under the Creative Commons Attribution 4.0 License [79].

A lung ultrasound (LUS) is a non-invasive imaging technique utilized to identify pulmonary abnormalities associated with ARDS, such as consolidations and edema. It exhibits high pooled specificity and intermediate sensitivity in diagnosing ARDS [78]. It can differentiate between two sub-phenotypes of ARDS, focal (characterized by dorsal inferior consolidations exhibiting overinflation with high positive end-expiratory pressure (PEEP) titration and/or recruitment maneuvers, thus indicating a need for prone positioning) and non-focal (marked by diffuse and patchy loss of aeration that responds positively to recruitment maneuvers), with excellent specificity and sensitivity [78]. Additionally, it can quantify the severity of lung injury in ARDS, achieving accuracies comparable to the current gold standard of chest imaging, computed tomography (CT) [78,79,80,81]. The utilization of LUSs is quickly escalating among individuals experiencing severe respiratory failure. A worldwide agreement to revise the ARDS definition was made in response to these developments [82]. A LUS 12 lung field-scoring system has been established, which relies on LUS aeration scores from both the left and right lungs in conjunction with the presence of anterolateral pleural line anomalies. It can precisely detect and rule out ARDS with external validation [83]. Figure 7 delineates the LUS scoring system. Lung regions exhibiting normal aeration (N) are assigned a score of 0; moderate aeration loss (B1) receives a score of 1; severe aeration loss (B2) is given a score of 2; and full aeration loss (consolidation; C) is allocated a score of 3. The cumulative readings for all 12 regions yield a LUS score ranging from 0 to 36. Reduced LUS scores indicate superior lung aeration, whereas elevated LUS levels signify worse aeration. A LUS score of 18 or above is associated with the necessity for intubation [84].

**Role of lung ultrasonography in the management of ARDS**

LUSs have recently been suggested as a method for guiding PEEP calculations. The LUS score facilitates the assessment of lung disease severity and enables the daily monitoring of lung aeration [84]. So far, limited research has assessed LUS-guided PEEP determination in patients with ARDS [80,81,83,85]. Among them, only one constitutes a randomized controlled study that compared ultrasound-guided PEEP determination with the standard-of-care technique of PEEP determination from the ARDSNet (Acute Respiratory Distress Syndrome Network) protocol [85]. In the experimental group, PEEP was established based on LUS scoring, with the lowest PEEP score corresponding to the lowest LUS score (showing optimal recruitment) as optimal. In the control group, PEEP was established in accordance with the ARDSNet protocol, identifying the PEEP value from the lowest FiO_2_–PEEP combination (as per the ARDSNet table) that sustained PaO_2_ between 60–80 mmHg or SpO_2_ between 88–95% as optimal [80]. The primary outcome of the trial was oxygenation, shown by the PaO_2_/FiO_2_ ratio. Upon trial completion, the LUS group had markedly elevated mean values for optimal PEEP, P/F ratio, static compliance, organ-dysfunction-free days, and ventilator-free days. It exhibited markedly reduced mean values for the Sequential Organ Failure Assessment (SOFA) score and the time of mechanical ventilation. The 28-day mortality in the LUS group was 6.7%, which was markedly lower than the 30.0% observed in the OXY group (*p* = 0.041). This study had a relatively small size and did not use ARDS sub-phenotypes in its analysis [81]. Further studies need to be conducted to validate these findings.

While LUS is well-validated in ARDS, there are currently no established studies or consensus on its application in CTD-induced DAH. Given this evidence gap, we propose a novel, hypothesis-driven framework for LUS-guided PEEP adjustment in CTD-DAH, adapted from ARDS-derived sonographic criteria but modified to account for the distinct pathophysiology of alveolar hemorrhage. Table 2 outlines these provisional recommendations, which are intended to serve as a pragmatic clinical approach until further studies can be performed.

Limitations of lung ultrasound in ARDS/CTD-induced DAH management

There are some limitations in employing lung ultrasonography for the management of ARDS/CTD-induced DAH, as outlined below:

- Lung ultrasonography is ineffective for identifying lung hyperinflation [81]. Nonetheless, few studies have evaluated the quantification of lung sliding and ultrasound elastography, which may assist in the assessment of lung hyperinflation [82,83,84,85];
- The lung ultrasonography score has a strong correlation with PEEP-induced elevations in end-expiratory lung volume, which reflects enhanced gas entry into already inflated lung regions rather than re-inflation. It correlates with tissue density and lung aeration, but not with alterations in the score resulting from variations in PEEP [85]. This is probably due to the score remaining unaffected by alterations in consolidation size or a reduction in the number of B lines [86];
- Information is contingent upon the operator and necessitates training for accurate interpretation.

#### 5.4.4. Extracorporeal Membrane Oxygenation (ECMO)

**Figure 8:**
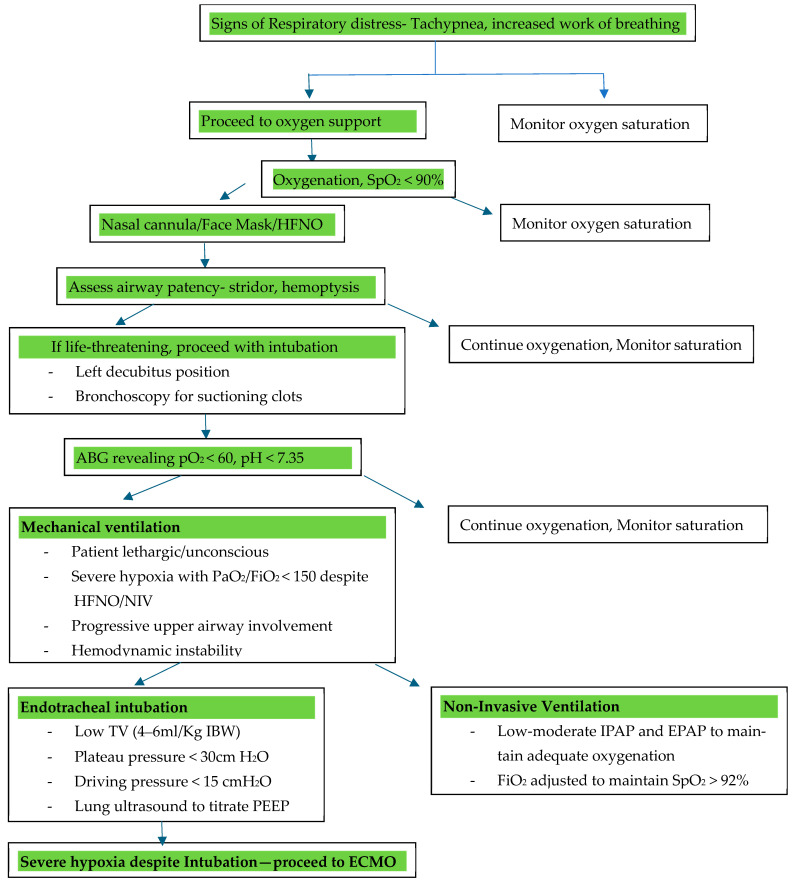
Flowchart of airway management in CTD-induced DAH. Abbreviations: SpO_2_: partial pressure of oxygen; HFNO: high-flow nasal oxygen; NIV: non-invasive ventilation; ABG: arterial blood gas; PaO_2_/FiO_2_: partial pressure of oxygen in arterial blood by the fraction of inspired oxygen; TV: tidal volume; IPAP: inspiratory positive airway pressure; EPAP: expiratory positive airway pressure; ECMO: extracorporeal membrane oxygenation; IBW: ideal body weight.

Extracorporeal membrane oxygenation (ECMO) has been used for patients with CTD-induced DAH, particularly when conventional treatments such as mechanical ventilation, immunosuppressive therapy, and supportive care fail to adequately address the severity of respiratory and hemodynamic compromise. Figure 8 depicts a flowchart on airway management in CTD-induced DAH, incorporating the use of ECMO.

Types of ECMO:

1. Veno-venous (VV) ECMO is used for respiratory failure with preserved cardiac function, such as in DAH, where massive pulmonary hemorrhage impairs gas exchange. It bypasses the lungs, supporting oxygenation and carbon dioxide removal. It can provide adequate gas exchange while allowing for lung-protective ventilation and mitigating ventilator-induced injury [83].
2. Veno-arterial (VA) ECMO provides both respiratory and cardiac support, typically used in cases of combined heart and lung failure, such as right heart failure secondary to severe pulmonary hypertension in CTD patients [83].

The Extracorporeal Life Support Organization (ELSO)’s 2021 guidelines recommend the following indications for VV ECMO in DAH [85]:

(1) Severe hypoxemia: PaO_2_/FiO_2_ < 80 mmHg despite optimal ventilation, including a trial of prone positioning;
(2) Hypercapnia: pH < 7.25 with a partial pressure of carbon dioxide in arterial blood (PaCO_2_ ≥ 60 mmHg), despite conventional mechanical ventilation (with a respiratory rate of 35 bpm and a plateau pressure ≤ 30 cmH_2_O);
(3) Development of ARDS due to widespread alveolar damage from DAH with failure of conventional ventilation [84,85].

The only absolute contraindication for the initiation of ECMO is assumed non-recovery without a plan for viable decannulation [85].

Ventilator settings with the use of ECMO

Ventilator settings are chosen to limit VILI while utilizing the extracorporeal circuit of VV ECMO [85]. Lung-protective settings (inspiratory plateau pressure (Pplat) < 25 cmH20, PEEP ≥ 10 cmH_2_O, respiratory rate (RR) 4–15 breaths/min (set RR) or spontaneous breathing, and FiO_2_ as low as possible to maintain saturations) are therefore utilized [85].

Generally, any ventilator mode (for example, pressure/assist control, volume/assist control, or airway pressure release ventilation) that can provide lung-protective ventilation during VV ECMO can be considered for ventilation [85]. Importantly, since oxygenation and carbon dioxide elimination are provided primarily by VV ECMO, the management should be via adjustments in the ECMO circuit and not by increasing the ventilatory settings [85].

Complications of ECMO in CTD-Induced DAH

The use of ECMO in patients with CTD-induced DAH presents several complications, many of which are related to the underlying autoimmune disease and its impact on hemostasis:

1. Hemorrhage: The most common and significant complication of ECMO in CTD-induced DAH is hemorrhage, primarily due to the systemic anticoagulation required to maintain circuit patency. Patients with CTDs are already at an increased risk of bleeding, particularly those with active disease and widespread vasculitis. The use of anticoagulants such as heparin increases the risk of bleeding within the lungs, worsening the alveolar hemorrhage and complicating management. Therefore, a delicate balance must be maintained between preventing clot formation in the ECMO circuit and avoiding further bleeding complications [86,87]. However, there have been some cases that are successfully managed without the use of anticoagulation, and other case reports utilizing systemic anticoagulation using a modified ACT target of 140 to 160 s [86,87].
2. Thromboembolism: Although less frequent, thromboembolic events can occur if clot formation occurs within the ECMO circuit [85]. This is a particular concern in patients who have an increased tendency to form blood clots due to autoimmune factors or the use of certain immunosuppressive medications [85].
3. Immunosuppressive Therapy and Drug Bioavailability: Patients with CTD-induced DAH typically require aggressive immunosuppressive therapy, such as corticosteroids, cyclophosphamide, or rituximab, to control disease activity and prevent further alveolar hemorrhage. However, the pharmacokinetics of these drugs in the context of ECMO remain poorly understood. ECMO itself can alter the bioavailability of medications, affecting drug absorption, distribution, metabolism, and elimination [86].
4. Infection: Local infections at cannulation sites or systemic infections related to the ECMO circuit are possible. The immunosuppressive treatment used to control CTDs may increase the susceptibility to infections, complicating the management of patients on ECMO [85].
5. Technical Issues: ECMO requires complex management of the cannulation sites and the circuit itself, and technical problems such as dislodged cannulas, circuit malfunctions, or air embolisms can occur. These issues may lead to complications that could affect patient outcomes [83,85].

Clinical outcomes of ECMO in CTD-induced DAH:

The clinical outcomes of ECMO in CTD-induced DAH have been mixed, and evidence is limited [88,89]. While ECMO has been shown to provide temporary respiratory support, it does not address the underlying pathophysiology of CTD-induced DAH. Therefore, the success of ECMO therapy in these patients depends heavily on the effective control of disease activity with immunosuppressive drugs. Further studies are needed to clarify the role of ECMO in improving survival outcomes for patients with CTD-induced DAH.

#### 5.4.5. Sedation and Pain Management

Airway management and mechanical ventilation require effective sedation and pain control. Sedation must be properly titrated to promote patient comfort and prevent respiratory depression. Propofol and dexmedetomidine are favored for their fast onset and low hemodynamic effect. Dexmedetomidine is recommended over propofol for light sedation in mechanically ventilated adult ICU patients (conditional recommendation, moderate certainty) by the 2025 PADIS guidelines [90]. The Intensive Care Medicine Rapid Practice Guidelines favor dexmedetomidine above alternative sedatives to reduce ICU delirium [91]. Using validated tools such as the Behavioral Pain Scale (BPS) and Critical-Care Pain Observation Tool, non-communicative and ventilated patients should have their pain management monitored routinely. The BPS and CPOT are the most valid and reliable pain monitors for this population [92]. Acetaminophen, nefopam, ketamine, lidocaine, neuropathic agents, and NSAIDs improve analgesic efficacy, but intravenous opioids remain the mainstay [92]. Multimodal analgesia and sedation with lower medication dosages can be achieved with opioids and sedatives/non-opioids, and localized anesthetic techniques such as thoracic epidurals can minimize systemic opioid requirements [93]. Drug selection in CTD patients must account for altered metabolism and increased sensitivity due to renal or hepatic dysfunction. Hemodynamic stability should be maintained, especially in pulmonary hypertension patients, and mechanical ventilation should be weaned off early to avoid sedation-related problems [94,95].

## 6. Monitoring and Follow-Up

Patients with acute hypoxic respiratory failure due to CTD-induced DAH require intensive care unit (ICU) admission and monitoring. Table 3 summarizes the monitoring and follow-up essential for recovery.

### Strategies for Follow-Up

Once stabilized, a structured follow-up plan is essential for avoiding relapses and improving long-term outcomes.

1. Imaging and Pulmonary Function Monitoring:
  - Chest X-ray or HRCT at regular intervals to assess DAH resolution;
  - Pulmonary function tests (PFTs) assess residual restrictive lung disease or diffusion impairment following recovery [17].
2. Immunosuppressive Therapy Optimization:
  - Depending on the CTD phenotype and severity, long-term immunosuppressive therapy, such as rituximab, cyclophosphamide, and mycophenolate mofetil, is used. A steroid-tapering strategy is used based on the clinical response [99].
3. Preventive Strategies and Rehabilitation:
  - Administration of vaccinations (influenza, pneumococcal, and COVID-19) to prevent relapse-inducing infections [103];
  - Pulmonary rehabilitation is reserved for patients with persistent dyspnea or deconditioning [104];
  - Early rheumatology follow-up is recommended to prevent relapses and control the disease [104].

## 7. Prognosis

Diffuse alveolar hemorrhage (DAH) is a life-threatening condition with an in-hospital mortality rate of approximately 20% [62,105]. The prognosis of CTD-induced DAH is highly variable and depends on several factors, namely the underlying CTD, the severity of hemorrhage, and the timeliness of interventions.

The early detection of DAH is critical to reducing the risk of respiratory failure and renal disease. SLE and ANCA-associated vasculitis are associated with higher mortality rates compared to other CTDs [106]. In patients with AAV-induced DAH, the severity of hypoxemia at presentation is the strongest predictor of respiratory failure [106].

Patients with rapidly progressive disease or severe respiratory compromise often require intensive care and mechanical ventilation. Delays in diagnosis or treatment can lead to irreversible lung damage, renal failure, and increased mortality [107]. However, individuals with DAH caused by systemic vasculitis, such as granulomatosis with polyangiitis (GPA) or microscopic polyangiitis (MPA), generally respond well to immunosuppressive therapies, including corticosteroids and cyclophosphamide, leading to more favorable outcomes.

## 8. Conclusions

The overall prognosis of DAH depends on early detection and timely intervention. Prompt recognition and aggressive management are essential to improving outcomes and reducing the risk of complications such as respiratory failure, renal disease, and death.

Respiratory management plays a critical role in the care of CTD-induced DAH patients, particularly in those with severe hypoxemia or respiratory failure. Strategies such as mechanical ventilation with lung-protective settings, high-flow oxygen therapy, and, in refractory cases, ECMO can be lifesaving. Our proposed novel airway risk stratification tool and LUS protocol (derived from ARDS principles and incorporating DAH-specific modifications) address the critical unmet need of a structured approach to managing CTD-induced DAH from emergency assessment to ventilatory optimization. Further studies are needed to validate these tools.

By integrating early diagnosis, immunosuppressive therapy, and effective respiratory support through a multidisciplinary team model, clinicians can significantly improve the prognosis of patients with CTD-induced DAH.

## Figures and Tables

**Table 1:** Serological tests for various connective tissue disorders that cause diffuse alveolar hemorrhage.

Connective Tissue Disorder	Specific Serological Labs	Non-Specific Labs
SLE	ANA, anti-dsDNA, anti-SM, anti	Increased ESR
	-histone antibody	
Anti-phospholipid antibody syndrome	APL, lupus anticoagulant,	Eosinophilia
	anti-CL, anti-β2GP1 antibody	
Rheumatoid arthritis	RF, anti-CCP	Hematuria
Polymyositis and Dermatomyositis	Anti-Jo-1	
Systemic sclerosis	Anti-topoisomerase I (Scl-70),	Anemia
	Anti-RNA polymerase III antibodies	
Mixed connective tissue disorder	Anti-RNP	
Granulomatosis with polyangiitis (GPA)	c-ANCA	Prolonged PT/aPTT/PT INR
Microscopic polyangiitis (MPA)	p-ANCA	Thrombocytopenia

Abbreviations: SLE: systemic lupus erythematosus; anti-dsDNA antibody: anti-double stranded DNA antibody; ANA: anti-nuclear antibody; anti-SM antibody: anti-smooth muscle antibody; ANCA: anti-neutrophil cytoplasmic antibody; anti-β2GP1: anti-β-2 glycoprotein1 antibody; c-ANCA: cytoplasmic anti-neutrophil cytoplasmic antibody; ESR: erythrocyte sediment rate; p-ANCA: perinuclear anti-neutrophil cytoplasmic antibody; PT: prothrombin time; INR: international normalized ratio; aPTT: activated partial thromboplastin time; RA: rheumatoid arthritis; RF: rheumatoid factor; anti-CCP: anti-cyclic citrullinated peptide; anti-RNP: anti-ribonucleoprotein antibodies; anti-Jo-1: anti-Histidyl-tRNA synthetase antibody; anti-CL antibody: anti-cardiolipin antibody; APL: anti-phospholipid antibody.

**Table 2:** Proposed adaptations of lung ultrasounds in ARDS for CTD-induced DAH.

Lung Ultrasound Findings	CTD-Induced DAH Adaptation	Clinical Decision-Making
Focal/non-focal sub-phenotypes		Non-focal: uniform PEEP
	Non-focal in most CTDs,	Focal: lower PEEP with
	Focal in GPA	lateral decubitus on
		bleeding side.
B-lines		Fibrin reduces compliance,
		leading to less PEEP
		Responsiveness—start lower PEEP
	Patchy/asymmetric due to blood	and adjust accordingly
		-SS-induced fibrosis can mask B-
		lines, adjust PEEP to avoid
		pneumothorax [28].
		-SLE/APS leads to increased
		vascular fragility, titrate
		PEEP cautiously [82].
Consolidations	Clotted blood	Avoid overdistension
		(barotrauma), lesser
		PEEP max, use ECMO early if
		worsening (described below).
Pleural line		CTDs can cause pleural
	Thickened/irregular	inflammation correlating
		with CTD activity—titrate
		immunosuppression. Effusions
		can reduce compliance, titrate
		PEEP and monitor for
		hemodynamic compromise.
Zones involved	Anterior/lateral	Blood pools in gravity
		-dependent areas early
Improvement in B-lines	Improving DAH	-Cautiously reduce PEEP
New B-lines/consolidation	Worsening DAH	-Assess for re-bleeding, titrate
		Supportive therapies, avoid increasing
		PEEP

Abbreviations: CTD: connective tissue disorder; PEEP: positive end-expiratory pressure; SLE/APS: systemic lupus erythematosus/anti-phospholipid syndrome; GPA: granulomatosis with polyangiitis; SS: systemic sclerosis; ECMO: extracorporeal membrane oxygenation; DAH: diffuse alveolar hemorrhage.

**Table 3:** ICU monitoring and follow-up for CTD-induced DAH.

Category	Key Component	Clinical Action
Respiratory monitoring	-ABGs -Serial HRCT -POCUS (lung aeration, volume status) -SpO_2_/EtCO_2_ (on ventilated patients) [96,97]	-Early intubation if PaO_2_/FiO_2_ < 200 -Lung-protective ventilation
Hemodynamic monitoring	-Arterial line for continuous blood pressure monitoring. -Fluid resuscitation guided by CVP measurement, POCUS [98]	-Vasopressor support for hemodynamic instability/shock -Avoid volume overload as can worsen pulmonary edema
Renal monitoring	-Daily creatinine/electrolytes -Urine output -Lupus nephritis/ANCA-GN screening [99]	-Nephrology consultation for AKI -CRRT if refractory acidosis /volume overload
Hematologic/Coagulation	-Hb, platelets, Fibrinogen, D-Dimer -TMA screening (SLE/APS) [100]	-PRBC transfusion if Hb < 7 g/dL -PLEX for ANCA vasculitis/catastrophic APS
Ventilation Strategy	-Low TV -Moderate PEEP -Permissive hypercapnia [73,74]	-Avoid barotrauma -ECMO if refractory hypoxia
Multidisciplinary Team approach	Address respiratory failure, CTD-induced immune dysfunction, and anatomical risks and provide longitudinal care. -Pulmonology: bronchoscopy, imaging -Rheumatology: immuno- suppression -Medical intensive care: ECMO/hemodynamics -Anesthesiology: address difficult airway -ENT surgeons: manage structural complications -Hematology: coagulopathy -Nephrology: AKI/CRRT -Respiratory therapy: ventilator weaning	-Daily interdisciplinary rounds [101,102] -Tailor therapy to CTD subtype (rituximab for AAV, anticoagulation for APS)

Abbreviations: ABG: arterial blood gas; HRCT: high-resolution CT; POCUS: point-of-care ultrasound; SpO_2_: partial pressure of oxygen; EtCO_2_: end tidal carbon dioxide; PaO_2_/FiO_2_: partial pressure of oxygen in arterial blood by the fraction of inspired oxygen; CVP: central venous pressure; AKI: acute kidney injury; CRRT: continuous renal replacement therapy; Hb: hemoglobin; TMA: thrombotic microangiopathy; PEEP: positive end-expiratory pressure; ECMO: extracorporeal membrane oxygenation; PLEX: plasma exchange; AAV: anti-neutrophil cytoplasmic antibody-associated vasculitis; APS: anti-phospholipid syndrome.

## Data Availability

Not applicable.

## Supplementary Materials

The following supporting information can be downloaded at: https://www.mdpi.com/article/10.3390/life15050793/s1, Table S1: Features of systemic and pulmonary involvement in connective tissue disorders associated with diffuse alveolar hemorrhage.
