# Supplementary material for: Connective Tissue Disorder-Induced Diffuse Alveolar Hemorrhage: A Comprehensive Review with an Emphasis on Airway and Respiratory Management
Source: Life (Basel). 2025 May 15;15(5):793. doi: 10.3390/life15050793 (PMC12113093; doi:10.3390/life15050793)
Supplement: Supplementary file 1 [file life-15-00793-s001.zip › life-3569633-supplementary.pdf]

Table S1: Features of systemic and pulmonary involvement in CTD-induced DAH.

**Connective tissue disorder**	**Systemic involvement**	**Other pulmonary manifestations**
Systemic Lupus erythematosus [18,19]	**Muco-cutaneous**: ACLE, SCLE, CCLE, DLE **Musculoskeletal**:Lupus polyarthritis, avascular necrosis **Hematological**:Anemia, neutropenia, leukopenia **Neuro-psychiatric**:Headaches, aseptic-meningitis,seizures, demyelinating syndrome ,movement disorders **Renal**: Lupus nephritis, interstitial nephritis, lupus-vasculopathy, vasculitis **Cardiovascular**:Pericarditis, myocarditis,Libman-Sachs endocarditis **GI**:Esophageal-dysmotility, mesenteric vasculitis, lupus enteritis, peritonitis, protein losing enteropathy, lupoid hepatitis **Ocular**: keratoconjunctivitis sicca, retinal vasculitis, optic neuritis, uveitis, scleritis, episcleritis	Pleuritis, Exudative pleural effusions,Acute-lupus pneumonitis,Non-specific interstitial pneumonia (NSIP), UIP, lymphocytic interstitial pneumonia, PAH, Pulmonary embolism,mediastinal lymphadenopathy
Rheumatoid arthritis [24,25]	**Articular:**Ulnar-deviation, metacarpophalangeal-joint subluxation,boutonniere deformity, **Extra-articular**: secondary Sjogren syndrome, Rheumatoid vasculitis, chronic non-healing ulcers	ILD, organizing pneumonia, PHTN, pleuritis, empyema,Rheumatoid nodules, pleural effusion
Anti-Phospholipid Syndrome [26,27]	**Skin**: Livedo reticularis, skin ulcerations, nail fold infarcts, digital-gangrene,necrotizing purpura **Hematological:**Thrombocytopenia **Neurological**: ischemic stroke, seizures, multi-infarct dementia **Cardiac:**Acute coronary syndrome **Renal:** hypertension, renal artery thrombosis	Pulmonary embolism, PHTN
Systemic Sclerosis [28]	Distal cutaneous sclerosis: below knees,Raynaud's	PHTN,ILD,NSIP,UIP,organizing pneumonia,Aspiration-pneumonia, pleural

	phenomenon,subcutaneous calcinosis, esophageal dysmotility, sclerodactyly, telangiectasis<br>Diffuse:Proximal skin involvement	involvement
Polymyositis and Dermatomyositis [29]	As part of anti-synthetase syndrome: Thick cracked skin over the tips and sides of the fingers (mechanics-hands), Raynaud's phenomenon.	ILD, Aspiration pneumonia, respiratory muscle weakness, PHTN, pleural involvement, organizing pneumonia, NSIP, UIP, lymphocytic interstitial pneumonia
Mixed Connective Tissue Disease [30]	**Skin**: Raynaud phenomenon, puffy-digits,scleroderma-like pattern to nailfolds, hand edema, acro-sclerosis<br>**Musculoskeletal**: Inflammatory arthritis, painless proximal muscle weakness<br>**Cardiac**: Pericarditis, mitral valve prolapse, myocarditis<br>**Renal**: membranous nephropathy<br>**Neurological**:Trigeminal neuralgia, peripheral neuropathy, aseptic meningitis<br>**Hematological:**Anemia, leukopenia, thrombocytopenia	ILD, pulmonary fibrosis, pleural effusion, PHTN, pulmonary thromboembolism, esophageal-dysmotility causing aspiration pneumonia
Granulomatosis with Polyangiitis (GPA) [20,21]	**Renal**: Rapidly progressive crescentic glomerulonephritis<br>**Ocular**: scleritis, conjunctivitis, peripheral ulcerative keratitis, pseudo-tumors<br>**Ear**: conductive and sensorineural hearing loss<br>**Skin:**Nodules,purpura, granulomas<br>**Neurological**:Peripheral neuropathy,pachymeningitis, seizure, cerebritis<br>**Cardiac**: Pericarditis, coronary arteritis, valvular insufficiency	Pulmonary nodules, pleural effusion, purulent nasal discharge, epistaxis, nasal ulceration
Microscopic Polyangiitis (MPA) [22,23]	Renal: Glomerulonephritis<br>Skin: Palpable purpura, livedo reticularis, urticaria, ulcers with necrosis<br>GI: Bleeding<br>Neurological:Peripheral neuropathy,patchy-meningitis, mono-neuritis multiplex	Chronic interstitial fibrosis

Abbreviations: ACLE: Acute cutaneous lupus erythematosus; SCLE: subacute cutaneous lupus erythematosus; CCLE: Chronic cutaneous lupus erythematosus; DLE: Discoid lupus erythematosus ; UIP: usual interstitial pneumonia;PAH: Pulmonary Arterial Hypertension; PHTN:Pulmonary hypertension; ILD: Interstitial lung disease ; NSIP: Nonspecific interstitial pneumonia.
